# Hidden figures: epistemic costs and benefits of detecting (invisible) diversity in science

**DOI:** 10.1007/s13194-021-00349-6

**Published:** 2021-03-03

**Authors:** Uwe Peters

**Affiliations:** 1 Center for Science and Thought, University of Bonn, Am Hof 1, 53113 Bonn, Germany; 2 Leverhulme Centre for the Future of Intelligence, University of Cambridge, Cambridge, UK; 3 Department of Psychology, King’s College London, London, UK

**Keywords:** Demographic diversity, Epistemic effects, Scientific groups

## Abstract

Demographic diversity might often be present in a group without group members noticing it. What are the epistemic effects if they do? Several philosophers and social scientists have recently argued that when individuals detect demographic diversity in their group, this can result in epistemic benefits even if that diversity doesn’t involve cognitive differences. Here I critically discuss research advocating this proposal, introduce a distinction between two types of detection of demographic diversity, and apply this distinction to the theorizing on diversity in science. Focusing on ‘invisible’ diversity (i.e., differences in, e.g., LGBTQ+, religious, or political orientation), I argue that in one common kind of group in science, if group members have full insight into their group’s diversity, this is likely to create epistemic costs. These costs can be avoided and epistemic benefits gained if group members only partly detect their group’s diversity. There is thus an epistemic reason for context-dependent limitations on scientists’ insight into the diversity of their group.

## Introduction

In philosophy of science, social epistemology, and the social sciences, there has been much work on diversity in social groups (e.g., Wylie 2006; Rolin 2017; Grim et al. 2019; Muldoon 2017; Eagly 2016; Page 2017; Peters 2020). One key question in that area of research concerns the epistemic effects of diversity, that is, the effects that diversity might have on belief formation and knowledge acquisition in social groups.

When considering the issue, researchers tend to distinguish between two kinds of diversity: *cognitive* diversity and *demographic* diversity. Cognitive diversity (henceforth *CD*)^1^ captures people’s differences in information, perspectives, experiences, education, or thinking styles, where all these differences are relevant for a given task. In contrast, demographic diversity (henceforth *DD*)^2^ captures people’s differences with respect to how they socially identify themselves and others (e.g., as female, African-American, mathematician, liberal, Jewish, etc.) (Page 2017: 169; Phillips 2017: 227; Steel et al. 2019: 4).

While CD and DD are distinct, they can overlap in ways that are important for investigating the epistemic impact of diversity. For instance, DD frequently results in CD because membership in different social groups (e.g., gender or race) often comes with different task-relevant information, perspectives, or experiences. Relatedly, while, for example, political diversity (say, whether an individual is liberal vs. conservative) is primarily an instance of DD (as it is tied to how individuals socially self-identify), it is also often an instance of CD, namely whenever it involves task-relevant informational differences. But political diversity isn’t always CD (as I shall use the terms here)^3^ because sometimes whether one is liberal, conservative, etc. is irrelevant to a given task (including normative research; Peters et al. 2020).

Focusing on task-relevant informational differences, many philosophers of science hold that it is precisely because DD and CD are commonly connected that DD can be epistemically beneficial: via adding cognitive differences to a group, DD can enhance social criticism, help counteract biased belief formation, increase creativity, facilitate a thorough exploration of problem space, and reduce research lacunas (Longino 2002: 132f; Solomon 2009; Fehr 2011; Rolin 2017: 118f; Peters 2019). That is, many philosophers take the epistemic gains tied to DD to be *mediated* by the corresponding CD (Steel et al. 2019).

While this is a common view in philosophy of science, several researchers have recently proposed an interesting alternative. They argue that in a group displaying DD, even if there is no corresponding CD, when group members notice or perceive their group’s demographic diversity (henceforth *DD detection*), they nonetheless start expecting CD and social similarity/dissimilarity with others. And these expectations, the argument continues, can significantly enhance information processing in the group (Carter and Phillips 2017; Phillips 2017; Steel et al. 2019). Call this proposal the *CD-independent view* of the epistemic benefits of DD.

Here I want to take a closer look at the CD-independent view. While I’m sympathetic to the proposal, I shall argue that in recent research on the CD-independent view – specifically in Carter and Phillips (2017) and Steel et al. (2019) – DD detection remains largely unanalyzed, and this results in unwarranted claims about the epistemic impact of diversity. To guard against such claims, two different kinds of DD detection should be distinguished, what I shall call *partial* and *specific* DD detection, which capture different degrees of insight into the distribution of DD within a group. Applying this distinction (further specified below) to the theorizing on diversity in groups in science, I contend that in one common kind of scientific group, if group members have full insight into the DD of their group, this is likely to create epistemic costs. I argue that these costs can be avoided and epistemic benefits gained if group members only partly detect the group’s DD. There is thus an epistemic reason for context-dependent limitations on scientists’ insight into the diversity of their group.

In arguing for these claims and analyzing DD detection, I will focus a type of DD that strikes me as intriguing but has so far been largely overlooked in philosophical research: While philosophers working on diversity have focused predominantly only on “visible” DD (e.g., gender or race diversity) or CD (Steel et al. 2019: 15; Longino 2002: 132f; Page 2017: 169; Pöyhönen 2017), there is also what some social scientists call “invisible” DD (Clair et al. 2005; Lambert and Bell 2013: 23f). It captures differences in social identity that are difficult to discern in face-to-face interactions, for instance, variance in sexual orientation (LGBTQ+), religious conviction, political ideology, socio-economics status, etc. While the term ‘invisible’ might be too strong, it is clear enough that unlike, say, gender or ethnicity, these differences can’t usually be swiftly read off, for example, from a subject’s physiology or name, and they are often deliberately concealed (Clair et al. 2005; Shields and Dunn 2016; Peters et al. 2020). Importantly, invisible DD isn’t the same as CD: group members’ invisible DD frequently involves differences in information, perspectives, and so on that are *irrelevant* to group tasks, whereas CD (as I use the term here) only captures task-relevant differences in information, perspectives, etc. (see also Page 2017: 169; Phillips 2017: 227). Since there is evidence that scientific groups often display at least some invisible DD (Ecklund et al. 2016; Shields and Dunn 2016; Barres 2018), if, as the CD-independent view suggests, DD detection can be epistemically beneficial, then this might provide steering committees of scientific groups with an epistemic rationale for making invisible features of DD in their groups more salient to group members. Exploring the effects of DD detection is thus important to determine whether it would promote or hinder epistemic success in science.

To make progress on this front, I begin in section 2 by introducing the key arguments that have been proposed for the CD-independent view. In sections 3 to 5, I then discuss recent research advocating the CD-independent view before distinguishing two kinds of DD detection. In sections 6 and 7, I apply the distinction to the theorizing on diversity in scientific groups and address potential objections to the approach to DD detection developed in the preceding sections.

Three clarifications should be mentioned. Firstly, I use terms such as ‘detecting’ and ‘noticing’ DD broadly: While they are typically factive, here their referents include merely perceiving or forming beliefs about DD, which needn’t be veridical or true, respectively. Thus construed, detecting or noticing DD (e.g., gender and race differences) needn’t always involve tracking pre-existing properties of individuals but might involve socially constructing or conferring them (Ásta 2018). By using terms in this way, I wish to remain neutral on the ontology of social identities (gender, race, etc.).

Secondly, when considering the epistemic costs and benefits related to DD detection, one needs to make assumptions about what ‘epistemic benefits’ are. This will depend on one’s preferred epistemic commitments (e.g., whether one is a virtue epistemologist, epistemic deontologist, or epistemic consequentialist). Here I adopt a veritistic version of epistemic consequentialism, according to which a practice *P* is epistemically beneficial in a particular way *W* for a subject/group *S* in context *C* if *P* facilitates the acquisition of true beliefs of interest for *S* in *C*.

Finally, notice that even if *P* is epistemically beneficial in some way *W* in *C*, this is not to say that it is epistemically *good* all-things-considered. For it might still be epistemically costly in other ways and other considerations (e.g., moral, prudential, etc.) might outweigh the epistemic benefit(s) of adopting *P* in *C*. Relatedly, the epistemic reason for context-dependent limitations on scientists’ insight into the diversity of their group that I shall introduce is defeasible; it might be overridden by other considerations. Having said that, as will become clear below, it is significant and should be taken into account when reflecting on how to design epistemically successful groups in science.

## Arguments for the CD-independent view

While many researchers suggest that DD needs to involve task-relevant cognitive differences to produce epistemic gains (Longino 2002: 130, 134f; Antony 2016; Page 2017: 169), on the basis of empirical studies, some social scientists and philosophers have recently maintained otherwise. For instance, Phillips and her colleagues (Phillips and Loyd 2006; Loyd et al. 2013; Carter and Phillips 2017) have argued that DD in a group can improve epistemic group performance without being connected to CD – at least as long as group members also *detect* the group’s DD. Phillips et al.’s arguments for this proposal, i.e., the CD-independent view, have also been related to the theorizing on diversity in philosophy of science (Steel et al. 2019). They will occupy center stage in the discussion on DD detection below. I will thus now briefly introduce Phillips et al.’s two main considerations and empirical support for the CD-independent view. In outline, they are as follows (see Phillips and Loyd 2006; Loyd et al. 2013; Carter and Phillips 2017):

1. Even in the absence of CD, when people detect DD in their group, this can be epistemically beneficial in generating expectations of cognitive differences that sharpen people’s information processing and prime them for new ideas. For instance, studies found that, compared to controls, white jurors in a racially diverse group raised more novel case facts, made fewer errors in their discussion of the case, and identified more missing data in the deliberation on a case than white jurors in homogeneous juries (Sommers 2006). Similarly, following group discussion on controversial issues, white subjects showed more sophisticated thinking in essay-writing tasks when assigned to a discussion group with a black minority-opinion holder than when assigned to an all-white group with a white minority opinion holder (Antonio et al. 2004). Moreover, subjects who anticipated entering a demographically homogeneous group meeting were found to prepare less thoroughly for the meeting than those anticipating a diverse group (Loyd et al. 2013). Interpreting the data, Phillips (2017) argues that subjects in homogeneous groups are less attentive to task-relevant information because they expect agreement and smooth interaction in the group whereas subjects in diverse groups expect disagreement and divergent views, prompting them to be more sensitive to novel information and to think more carefully.

(2) Even in the absence of CD, when people notice DD in their group, this can be epistemically beneficial in that it reduces group members’ unwarranted attributions of credibility and trust to identity-similar others while also making them more tolerant of disagreement, more confident about articulating dissent, and more focused on a given task. The reason has to do with “similarity-attraction”: studies suggest that people are attracted to, seek alignment with, and expect agreement from similar subjects, which can incline them to take identity-similar group members to be more credible and trustworthy than identity-dissimilar members even when this is unjustified (Carter and Phillips 2017). In homogenous groups, this process can make subjects reluctant to express dissenting views, which then contributes to epistemic in-group conformity. In contrast, in diverse groups, people are less affected by similarity-attraction, and this makes them more willing to express disagreement and to focus on the task, as they are less concerned about social bonding. Experiments found, for instance, that in a group of students, when a member of the demographic majority in the group expressed a different view than the rest, in homogeneous groups, subjects displayed a stronger aversion against disagreement and engaged less in a given task than in diverse groups (Phillips and Loyd 2006). That is, diverse groups showed greater task engagement and facilitated more persistent and confident expressions of disagreements.

While (1) and (2) provide plausible motivations for the CD-independent view, in the remainder I shall argue that some recent research that is built on these points is problematic. I begin by scrutinizing philosophical work by Steel et al. (2019) before critiquing Phillips et al.’s own account of diversity based on (1) and (2). The rationale for and goal of the following critical discussion is not to provide conclusive objections to other researchers’ work on the CD-independent view but to help take it further and elucidate the nature of epistemically beneficial DD detection.

## From the epistemic benefits of DD to epistemic injustice

In philosophy of science, Steel et al. (2019) argue that even without CD, due to “cognitive diversity expectation” (i.e., the mechanism described in (1)) and “in-group epistemic conformity” (i.e., the mechanism described in (2)), DD can be epistemically beneficial in science by contributing to “information elaboration”, the “process whereby knowledge dispersed in a group is elicited and examined” (8). Steel et al. maintain that (a) several philosophical accounts of diversity have so far overlooked this possibility, i.e., that DD may improve group performance independently of CD. Moreover, Steel et al. hold that (b) this CD-independent effect of DD suggests a “potential divergence of epistemic and equity-based rationales for diversity” (2019: 16). I agree with Steel et al. on (a) and shall set it aside. I want to focus on (b). Steel et al. maintain that the two mechanisms that in their view account for the CD-independent benefits of DD, namely CD expectation and in-group epistemic conformity involve epistemic injustice, and so epistemic benefits of diversity can be epistemically unjust. Steel et al. support their claim with two arguments. One of them provides me with a helpful starting point for analyzing the cognition that is involved in epistemically beneficial DD detection. I will thus now briefly introduce and assess it.

Steel et al.’s (2019: 14f) argument can be summarized as follows:

(P1) DD in a group can lead group members who detect it to expect CD, and this CD expectation is epistemically beneficial in priming the group members to potential intersubjective disagreements, causing them to assess information more carefully and be more susceptible to new ideas.

(P2) The CD expectation mechanism (wherein group members transition from DD detection to CD expectation) involves a “tendency to stereotype in which noticeable markers of social identity are treated as accurate predictors of beliefs, knowledge, perspectives, and so on” (Steel et al. 2019: 15).

(P3) Such stereotyping can be epistemically unjust (ibid: 16).

(C) So, DD can result in epistemic benefits that can be epistemically unjust.

Steel et al. take the support for (P1) from Phillips et al.’s empirical work. And they add (P2) as an intuitively plausible assumption without providing further support.

There is reason to be cautious about this assumption, however. For instance, Carter and Phillips (2017) note that while the CD expectations underlying the epistemic benefits of DD do rest on “social categorization whereby individuals make in-group and out-group distinctions”, the social categorization involved can take two different pathways (8). On one pathway, Carter and Phillips argue, it activates “stereotypes” and “intergroup bias”, i.e., the tendency to display in-group favoritism vs. out-group derogation, which is likely to happen in competitive environments, when group identity is threatened or when strong social ‘fault-lines’ persist, i.e., when group members align along demographic characteristics causing a group to divide into homogeneous subgroups (ibid). Crucially, when social categorization is taking this “first pathway”, then detrimental group processes marked by a “lack of trust”, “failure to adequately attend” to out-group members, and “interpersonal conflict, avoidance behaviors, and communication problems will emerge and ultimately undermine group performance”, Carter and Phillips (2017: 8-9) hold. They argue that it is only on the “second pathway”, which, on their view, involves social categorization that does *not* implicate stereotyping, that DD detection triggers expectations of CD that strengthen epistemic performance: it is only then that these expectations also involve the type of motivational mindset that prompts people to uncover and appreciate unique information, conduct a careful analysis, and invest effort in perspective taking when processing claims (ibid).^4^ These prima facie plausible points don’t yet undermine Steel et al.’s assumption that stereotyping *can* produce the epistemic benefits appealed to in (P1). They do, however, indicate that this assumption is in need of support.

Consider now (P3). Steel et al. (2019: 16) make (again) only an existential claim: in some cases, stereotyping can be an ‘epistemic injustice’, which they specify by referring to Pohlhaus (2017: 21), who writes that stereotyping is an epistemic injustice if it involves (a) “pernicious stereotypes”, (b) a “potential unfair bias through which [subjects’] epistemic activities may be received”, or if it (c) “impede[s] their epistemic activity in comparison to those who are not perniciously stereotyped”. Notice that if stereotyping involves only treating “noticeable markers of social identity as accurate predictors of beliefs, knowledge, perspectives, etc.” (Steel et al. 2019: 15), then that by itself does not yet link it to (a), (b), or (c), i.e., to an unequal, unfair, or unwarranted trait ascription. Call a tendency to treat certain noticeable markers of social identity automatically as accurate predictors of certain beliefs, etc., *neutral stereotyping*. Call a tendency that is the same but additionally also involves (a), (b), or (c) (and might implicate positive stereotypes) *loaded stereotyping.*

Given this distinction, what about Steel et al.’s claim that in the cases when the CD-expectation mechanism involves epistemically unjust, that is, loaded stereotyping, it can still lead to the kind of epistemic benefits mentioned in (P1)? Steel et al. rest content with the intuitive plausibility of the point. Yet, there is reason to doubt it. For instance, in a set of studies, van Dijk et al. (2018) found that “information elaboration” in diverse teams (*N* = 97), which Steel et al. explicitly take to be epistemically beneficial per se and important to preserve (see Steel et al. 2019: 6f), had itself *negative* epistemic effects in groups with inaccurate competence attributions: the disparately high influence of subjects who were inaccurately viewed as more competent inhibited group performance as the information-elaboration process focused on less reliable input than when accurate social perceptions were involved. Importantly, the competence attributions in van Dijk et al.’s study were based on loaded “stereotypes”: on “expected higher performance from women working on emotional tasks compared to math tasks, while for men this pattern was reversed” (2018: 8). The data thus challenge Steel et al.’s claim that when DD detection involves stereotype-based unequal, unfair, or unwarranted trait (e.g., trustworthiness) ascription to out-group vs. in-group individuals, this might produce epistemic benefits related to information elaboration. Van Dijk et al.’s findings put pressure on this view because they suggest that inaccurate attributions of low credibility to stereotyped individuals weakened group performance by contributing to group members’ oversight of potentially corrective contributions offered by those stereotyped.

The data don’t yet preclude Steel et al.’s claim that DD detection implicating stereotyping *can* be both epistemically unjust and epistemically beneficial. But since Steel et al. haven’t offered argumentative support for this claim^5^ and Van Dijk et al.’s findings provide reasons to doubt it, there is ground to remain skeptical that when DD detection triggers CD expectations based on loaded stereotypes, the epistemic benefits appealed to in (P1) might still arise. That is, Steel et al.'s view that DD can result in CD-independent epistemic benefits that are also epistemically unjust remains insufficiently supported.

To further investigate the nature of epistemically beneficial DD detection and its link to stereotyping and social categorization more generally, I will now consider Phillips et al.’s proposal on how DD detection yields CD-independent epistemic gains. As it turns out, this proposal too is not unproblematic.

## Is social categorization necessary for epistemically beneficial DD detection?

Carter and Phillips (2017) “argue that social categorization and resulting forces of similarity attraction are *necessary prerequisites* for eliciting beneficial information and decision-making processes that enhance group performance [emphasis original]” (9). Importantly, for Carter and Phillips, in each particular group in which DD has CD-independent epistemic benefits, the required social categorization in the group is one “whereby individuals make in-group and out-group distinctions so that out-group members are viewed as more different from the self, and in-group members are seen as more similar” (ibid: 6). That is, individuals of a diverse group need to classify specific group members in terms of their specific in-group vs. out-group features via “salient characteristics such as age, race, gender, and organizational membership, as well as other dimensions that may be meaningful within the given social context” (ibid: 3).

Carter and Phillips’ proposal seems natural. It arguably also underlies Steel et al.’s view that CD expectations based on DD detection are “plausibly explained by a human tendency to stereotype” others (2019: 15); after all, stereotyping is one form of social categorization. Steel et al. don’t hold that social categorization is *necessary* for the CD-independent epistemic benefits of DD, however. But independently of whether social categorization is assumed to be necessary for or just to plausibly explain the CD-expectation mechanism, I shall now argue that there is an alternative basis of CD-expectations and the related epistemic benefits of DD. Specifically (*pace* Carter and Phillips), for CD-independent, DD-detection related epistemic benefits to arise, group members don’t need to identify each other in terms of their specific demographic characteristics and draw individual specific similarity vs. dissimilarity distinctions among each other. Relatedly, the emergence of epistemically beneficial CD-expectations can plausibly be explained *without* assuming an involvement of loaded (or neutral) stereotyping of specific individuals. 

To see this, consider a group of people who display what I earlier called ‘invisible’ DD, that is, they exhibit demographic differences that are difficult to recognize in face-to-face interactions and result from self-identification (or social construction) in terms of sexual orientation, religion, political identity, socio-economics status, academic discipline, etc. (Clair et al. 2005; Lambert and Bell 2013: 23f). Suppose that while group members are unaware of the group’s invisible DD, the group leader, *L*, knows of it. *L* then informs the group that the group is demographically diverse in the mentioned respects but doesn’t yet tell them about the specific distribution of that diversity in the group. So *L* doesn’t tell the group about each individual’s particular sexual orientation, religion, political identity, and so on. Assuming that the group members accept *L*’s claim about their group’s composition, what are the likely epistemic effects?

While they now know that they are in a group with significant DD, the group members can’t categorize or stereotype each other with respect to the particular demographic differences just mentioned. They lack the specific distributional information. Correspondingly, when interacting with each other, they won’t be able to draw an individual-specific similar vs. dissimilar distinction with respect to the demographic dimensions at issue (though other dimensions, e.g., gender or ethnicity, might still be accessible for that purpose). And while the group members might, independently of what *L* told them, be looking for subtle cues to identify ‘who-is-who’ in terms of the demographic features at issue, they won’t be certain in each particular case. Since the subjects involved can’t confidently tell the relevant differences among each other, the insight into these differences provides them with little basis to be more socially drawn to or repelled from some specific group member but not others. Thus, with respect to the differences at issue, processes of individual-specific social categorization, stereotyping, and similarity-attraction are suspended.

Yet, among group members, there should still be a reduced concern about their social relationships and so greater task-focus as well as an increased expectation of disagreement, contributing to an uncovering of unique information, careful analysis, and effort in perspective taking, boosting problem-solving and creativity. This is because each group member now knows that the group as a whole is demographically diverse and so they have reason to expect differences in opinion in social interactions. The CD-independent epistemic benefits of DD mentioned above should thus still arise. After all, for all that each individual can tell the subject they are interacting with might be either an out-group or an in-group member with respect to the demographic features at issue. Since they can’t tell either way, the uncertainty induced should diminish subjects’ relationship focus, increase their attention to the task, and prime them to potential disagreements, sharpening their information processing.

There is empirical support for this. Levine et al. (2014) asked subjects to engage in (fictional) real estate trading with each other online. Subjects were randomly assigned to either an ethnically homogenous or an ethnically diverse market, i.e., group. Importantly, subjects knew of the ethnical homogeneity (in one condition) or ethical diversity (in another condition) in their groups but, while trading, couldn’t see each other and didn’t know which trader made a certain bid or offer. Subjects in the diverse-group condition were thus in the kind of context outlined in the hypothetical scenario above. They knew (invisible/task-irrelevant) DD was present, but didn’t know who belonged to which demographic group.

Interestingly, in the homogenous-group condition, even those with superior pricing skills were likely to commit pricing errors, buying and selling above true value, and traders were more likely to accept speculative prices. In contrast, subjects in the diverse-group condition reliably priced assets closer to true values (“market prices fitted true values 58% better”); they were less likely to accept inflated offers and more likely to accept offers that were closer to true value (Levine et al. 2014: 1). These data suggest that in diverse groups, the kind of DD detection envisaged above resulted in enhanced deliberation and epistemic benefits of the type that Phillips et al. and Steel et al. highlight.

Now, these effects might be explained (as Levine et al. 2014 do) by holding that in homogenous (vs. diverse) groups subjects place greater and unwarranted trust in similar others (vs. dissimilar others, respectively). Notice, however, that the study didn’t measure trust attributions, and it is equally plausible that the effects arose because subjects in diverse groups anticipated competition^6^ and assumed that their interactants were more knowledgeable than them, for instance, due to their different viewpoints.^7^ Either way, the results clearly suggest that (1) social categorization of specific individuals that one is interacting with is not required for the kind of CD-independent epistemic benefits discussed, and that (2) their emergence can be plausibly explained without invoking stereotyping.

To further motivate the view that the benefits at issue aren’t necessarily dependent on being less attracted to or trusting in dissimilar vs. similar others, consider again the hypothetical example from above involving group leader *L*. Suppose that, unlike before, now the group at issue consists of subjects who are equally affectively nonchalant towards demographically dissimilar vs. similar members. Suppose too that while the group members believe that demographic differences influence people’s information processing and behavior, they are unaware that their group is highly demographically diverse. Suppose finally that, as before, *L* then informs the group members that their group is vastly demographically diverse. But they again can’t detect the specific distribution of that diversity among each other.

Since they believe that DD influences people’s information processing and behavior, in subsequent interactions, each group member is likely to display an increased task-focus while also being primed to task-relevant differences. For unlike before when they were completely ignorant of the DD at issue, group members now have a positive reason to expect dissent and become the target of criticism – a position one arguably doesn’t want to be in no matter who is the source of the critique. Since these subjects are now by assumption equally affectively indifferent towards demographically dissimilar vs. similar members, the resulting epistemic benefits related to enhanced task-focus and sensitivity to task-relevant differences are independent of similarity-attraction.

This challenges Carter and Phillips’ (2017) proposal that social categorization involving similarity-attraction is necessary for the type of CD-independent epistemic benefits of DD at issue here. Because even though the preceding considerations are subject to future empirical testing, they are prima facie plausible. Given the absence of counterevidence, there is thus a basis for assuming that the relevant epistemic benefits don’t require social categorization in which a subject views herself as similar/dissimilar from her interactant(s) and can occur, and be plausibly explained, without such a categorization, including stereotyping.

## Two types of DD detection

The discussion of Carter and Phillips’, and Steel et al.’s views on the features of epistemically beneficial DD detection is instructive. It provides a rationale for distinguishing two different types of DD detection:

1. *partial DD detection*, which occurs when group members learn that their group is demographically diverse in various ways but still lack an insight into the specific distribution of that diversity among individual group members (i.e., they can’t categorize each other with respect to the relevant demographic aspects); and
2. *specific DD detection*, which occurs when group members learn that their group is demographically diverse in various ways and they also have an insight into the specific distribution of that diversity in the group (i.e., they can categorize each other with respect to the relevant demographic aspects).

Both (1) and (2) should be distinguished from (i) the presence and (ii) the use of DD within a group. For DD might be present in a group and the members of that group might register and use information from demographically diverse sources in their group (e.g., for the purpose of solving a problem) without yet being aware that the group is indeed demographically diverse. This point is highlighted by the fact that the diversity at issue might be invisible.

The distinctions between (1) and (2) and between the *presence, use,* and *detection* of DD in a group help refine the investigation of the epistemic impact of diversity. They enable us to ask, for instance, whether and, if so, how the impact of the presence of DD in a group is affected by group members’ noticing that the group is demographically diverse. We can thus distinguish *first-order* questions about the epistemic impact of diversity, that is, questions about the epistemic effects of processing information from diverse sources, from *second-order* questions, questions about the epistemic effects of processing information about a group’s diversity itself. Keeping these two types of questions apart and exploring the interplay between the types of effects that they pertain to will be important to attain a comprehensive picture of the epistemic impact of diversity.

Notice too that given the difference between the two kinds of DD detection, DD in a group might result in different kinds of epistemic effects depending on what type of DD detection occurs. One of them might be more epistemically beneficial in one context or group than in another. The distinction should thus be kept in mind when exploring the epistemic impact of diversity. To illustrate its explanatory fruitfulness further, I will now apply this distinction in the theorizing on the epistemic effects of DD in groups in science.

## DD detection in scientific groups

To make the discussion more tractable, I shall focus on a particular kind of group of scientists, namely groups of scientists who

- have been brought together to collaborate by a steering committee,
- don’t yet have extensive experience working in demographically highly diverse environments,
- aren’t yet familiar with each other, and
- display various aspects of DD that is task-irrelevant and invisible but known to the group’s steering committee.

So while the selected scientists do also vary in visible DD (e.g., gender and race), they mostly differ in that they belong to, say, different sexual (e.g., LGBTQ+), religious (e.g., Christian vs. atheist), and political (e.g., liberal vs. conservative) orientations as well as academic disciplines (e.g., a scientist with a degree in philosophy). And all these aspects of their social identity are both difficult to notice by each group member and not pertinent to the performance of the task that the group is given.

As noted earlier, scientific groups often do contain at least *some* degree of such invisible DD (see, e.g., Ecklund et al. 2016; Shields and Dunn 2016; Barres 2018). Especially in international research institutions focusing on grant-based, fixed-term projects investigating scientific problems that lend themselves to interdisciplinary approaches, groups of the kind described are likely to be common. Given the increasing calls for a general diversification of academia, they are likely to become more common in the future (e.g., Medin et al. 2017; Stewart and Valian 2018). For ease of exposition, I shall refer to groups meeting (a)-(d) as *demographically diverse scientific groups* (*DDS* groups) for short.

Since the members of DDS groups don’t yet notice most of their group’s diversity, what would be the epistemic effects if their steering committee facilitated either specific or partial DD detection within the group? To address the question, I shall develop the following argument:

(P1) In DDS groups operating in social condition *C*, which involves social environments in which individuals’ social expectations are shaped by competition, threats to group identity, and/or strong social fault-lines, *specific* DD detection is likely to create significant epistemic costs whereas *partial* DD detection helps avoid these costs and can also result in epistemic benefits.

(P2) DDS groups often operate in *C*.

(C) So, DDS groups often operate in a condition in which specific DD detection is likely to create significant epistemic costs whereas partial DD detection helps avoid these costs and can also result in epistemic benefits.

Notice that the argument is neutral on whether partial DD detection is overall, all things considered, more epistemically beneficial than specific DD detection. It is compatible with the view that the overall benefits (epistemic or otherwise) of specific DD detection outweigh those of partial DD detection. Despite these qualifications, if correct, the argument will still provide one hitherto unexplored epistemic reason in favor of adopting partial rather than specific DD detection in DDS groups that should be considered when theorizing about the epistemic impact of diversity in science.

What, then, is the support for (P1) and (P2)? As for (P1), Phillips et al.’s points (1) and (2) (from section 2 above) provide plausible reasons for holding that DD detection can produce significant CD-independent epistemic benefits by giving rise to CD expectations. A steering committee in charge of DDS groups has thus epistemic grounds to consider facilitating DD detection in their groups.

Recall too, however, that in social environments with competition, threats to group identity, and/or strong social fault-lines, *specific* DD detection is likely to lead to negative epistemic results. For the social categorizations that in such a situation (i.e., in social condition *C*) underlie people’s CD expectations is likely to activate intergroup bias, inclining group members to display a lack of trust and failures to adequately attend to out-group members, contributing to interpersonal conflict, avoidance behaviors, and so on. All of which weaken the epistemic performance of the group (see section 3). A DDS-group steering committee has thus epistemic reasons to avoid facilitating specific DD detection in *C*.

Importantly, however, a group in *C* need not yet forego the CD-independent epistemic benefits tied to DD detection. This is because, as argued above, for these kinds of benefits to arise, *partial* DD detection can be sufficient (e.g., Levine et al.’s (2014) study provided empirical support for this and offered a concrete example). And crucially, partial DD detection doesn’t yet involve enough insight into the social identity of group members to allow for an individual-specific in-group vs. out-group distinction. Hence, facilitating partial DD detection in DDS groups in *C* can allow a steering committee to reap DD-related CD-independent epistemic benefits without incurring the epistemic costs of specific DD detection. This supports (P1).^8^

Turning now to (P2), in what kind of social environment(s) do DDS groups operate? Two points are worth noting. Firstly, it is well known that scientists frequently compete for funding, recognition, and authority (Fang and Casadevall 2015). And while competition isn’t negative per se (e.g., it can help keep individual scholars’ confirmation bias in check; Ridley 2012), many researchers have noted that the sciences currently often involve “over-competitiveness” (Carson et al. 2013: 184), which has adverse effects “undermining resource sharing, research integrity, and creativity” (Fang and Casadevall 2015: 1229).

Secondly, there is evidence that scientists often display pronounced intergroup biases creating strong social fault-lines. For instance, Kirby et al. (2019) explored the “perceptual barriers” to scientific collaboration in teams crossing the boundary between the natural sciences and the social sciences. They found that, for instance, earth scientists (*N* = 449) “perceived social science/scientists as significantly less competent than natural science/scientists” (Kirby et al. 2019: 1). Similarly, Urbanska et al. (2019) surveyed 280 scientists and found that “in line with the intergroup bias literature, group membership in the more prestigious hard sciences is related to a stronger tendency to downplay the intellectual contribution of social science disciplines compared to other hard science disciplines” (1).

It is also well known that scientists often display intergroup biases when it comes to, for instance, race, gender, sexual, political, and religious orientation. For example, studies found that many scientists in the US are biased against Black and Latin-x post-doctoral candidates for STEM research positions (Eaton et al. 2019: 127). And in the STEM sector, LGBTQ+ researchers sometimes face aversive reactions from their straight colleagues (Cech and Pham 2017). Indeed, some data indicate that about 1/3 of physical scientists from sexual and gender minorities (i.e., LGBTQ+) in, for example, the UK have “considered leaving their jobs because of their workplace climate” (Gibney 2019: 16). Surveys also found that many scientists display an explicit willingness to discriminate against peers belonging to certain Christian groups (Yancey 2011; Barnes et al. 2020). And in several studies, both politically liberal and conservative researchers in the social sciences and humanities reported hostility and bias against colleagues with the opposite political orientation (Inbar and Lammers 2012; Shields and Dunn 2016; Honeycutt and Freberg 2017; Peters et al. 2020).

In sum, there is ground to suspect that scientists often operate under conditions of competition and harbour various inter-group biases against other members of their own and other fields. That is, individuals in scientific groups often operate in condition *C*. Since there is little reason to believe that DDS groups are different in this respect, in the absence of counterevidence, we have an inductive basis to assume (P2).

Both (P1) and (P2) are now in place and so (C) is supported. That is, DDS groups often operate in a condition in which specific DD detection is likely to create significant epistemic costs whereas partial DD detection can avoid these costs and also produce epistemic benefits. The argument for (C) provides steering committees of DDS groups with an epistemic reason for facilitating partial DD detection and limiting the opportunities for specific DD detection in the groups. Call this the *partial DD detection approach* to diversity.

## Four objections and clarifications

### Objection 1

Focusing on a key assumption underlying the partial DD detection approach, whether or not DD detection in DDS groups will generate epistemic benefits remains an open empirical question. This is because not just any DD is likely to trigger the type of mechanisms leading to epistemic benefits discussed, including expectations of CD and disagreement. The kinds of DD that are of interest are only the ones that have a significant impact on people’s social experiences and so provide a reason to expect CD and disagreement. Furthermore, the studies mentioned in section 2 pertain only to *visible* diversity and the partial DD detection approach concerns *invisible* DD. Hence, they provide little support for this approach.

### Response

While we don’t yet have relevant data specifically on DDS groups, some studies suggest that even the detection of aspects of invisible DD that are irrelevant to social experience and CD can produce the type of epistemic benefits at issue here: Phillips and Loyd (2006) told students that they would be working in teams of three to make a decision about the best company for another company to acquire. In one condition, subjects were informed that the two other team members were living on the same north/south side of campus as them (homogenous group condition). In another condition (diverse group condition), subjects were informed that only one was living there. Surprisingly, simply knowing of shared north/south accommodation location caused many subjects to expect viewpoint-similarity in the entirely *unrelated* task of deciding which company was best to acquire. And when a member of the demographic majority expressed a different view, in homogeneous groups, many subjects also had more negative feelings toward disagreement and engaged less in the task than in diverse groups. Since in this study an invisible aspect of diversity (i.e., subjects’ living north vs. south of campus) was made salient that has arguably a minor, if any, impact on people’s social experiences and CD, the experiment provides evidence that DD detection of that type can still produce the kind of CD-independent epistemic benefits discussed. The study by Levine et al. (2014) reviewed above offers another example. It too found that the partial detection of invisible DD (i.e., when individual-specific ethnicity was unknown) yielded epistemic benefits without that diversity being relevant to the task (i.e., real-estate trading). Since we have little reason to assume that in DDS groups, the results will be different, these data help address *Objection 1*.

### Objection 2

The partial DD detection approach is rarely relevant and hardly feasible. For instance, in most scientific groups, the social categories of group members (gender, race, etc.) are typically already well known to others, or become quickly salient in face-to-face interactions: in most scientific groups, even when it comes to invisible DD in DDS groups, scientists are likely to inadvertently signal their invisible social identity to their collaborators via the content of their contributions, style, conduct, language, etc.

### Response

Even with respect to visible features such as sex and race, recognition of the related demographic differences isn’t always easy. There are, for instance, trans-women/men or subjects with different multi-racial backgrounds whose distinctive demograpic or social category is difficult to perceive. Additionally, just as people in general, scientists often naturally differ along dimensions of invisible DD (e.g., sexual orientation, political viewpoint, etc.), and increasingly collaborate on research without having met in person; for instance, via online platforms or social networks (see also Zoom and the Covid-19 context) where various cues of social identity salient in personal interactions can more easily go unnoticed (Code and Zaparyniuk 2009; Van Noorden 2014; Gilson et al. 2015). Furthermore, while members of DDS groups might reveal their invisible social identities (and find out about each other’s demographic differences) via their contributions to the group, often group members won’t be certain about each other’s sexual orientation, religious or political identity, and so on: if these DD dimensions were easily detected, they wouldn’t be considered dimensions of *invisible* diversity in the first place. And, importantly, there is evidence that many scientists deliberately and successfully conceal aspects of their invisible social identities (Shields and Dunn 2016; Barres 2018; Peters et al. 2020). To precisely the same extent that invisible DD is real, namely frequently, partial (vs. specific) DD detection is not only relevant but also feasible. For there will then frequently be cases when steering committees of DDS groups may either leave hidden some DD dimensions that are currently not detected by group members, or make them more salient.

### Objection 3

The partial DD detection approach suggests that, in some cases, in DDS groups, scientists’ opportunities for specific DD detection should be limited. This, however, is unacceptable: some scientists might want to be open about and have others notice, for instance, their LGBTQ+, religious, political, etc. identity. Attempting to prevent this interferes with their basic right of self-expression.

### Response

The partial DD detection approach is primarily about whether or not to take measures to remove an existing lack of awareness of diversity within groups either moderately (i.e., partial DD detection) or extensively (i.e., specific DD detection). It is not necessarily also about whether to deprive group members of an otherwise for them easily attainable awareness of their diversity – let alone prevent them from expressing their social identity if they wish to do so. Having said that, suppose the members of a DDS group are informed about the above-mentioned considerations on the epistemically negative effects of social categorization in the context of DD detection. It isn’t unreasonable to assume that these scientists may then themselves, aware of the pernicious effects of their own implicit biases and stereotypes, want to adopt policies at work that help them avoid revealing their own invisible individual-specific social identities and detecting each other’s. As part of that effort, they might ask the group’s steering committee for assistance. This may result in their adoption of the partial DD detection approach and in limitations of their opportunities for specific DD detection that are fully compatible with respecting the scientists’ right to express (and be perceived as) who they are. Only these and related consent-involving cases of limiting scientists’ insights into their group’s diversity are at issue here.

### Objection 4

Hindering scientists in DDS groups from full disclosure of their invisible social identities so as to undercut the epistemic costs of specific DD detection is both unnecessary and potentially counterproductive. For the problem of intergroup biases that the partial DD detection approach is meant to avoid could alternatively and simply be solved by introducing social norms in DDS groups that encourage tolerance towards different sexual orientation, religious, political etc. identities among scientists. Indeed, preventing the detection of these identities reduces scientists’ opportunities to develop this tolerance at their work place: to become more tolerant, one needs to be exposed to and notice different social identities so as to receive corrective, stereotype-disconfirming evidence. The partial DD detection approach thus seems to undermine attempts to fight biases and stereotypes.

### Response

Unfortunately, specific social norms encouraging tolerance and social justice in DDS groups are perhaps unlikely to effectively keep, for instance, implicit biases and stereotyping in check. This is because these cognitions operate largely outside a subject’s awareness and direct control. Adding to the problem, with respect to many of the demographic dimensions at issue here, for instance, one’s political orientation, such norms would perhaps also be in tension with the moral framework that scientists encounter outside of academia. For example, while bias and discrimination on the basis of gender or ethnicity are in Western societies criticized – indeed illegal – when ‘hard’ scientists disrespect ‘soft’ scientists (Kirby et al. 2019), or when liberal scientists are hostile towards their conservative fellows and vice versa (Honeycutt and Freberg 2017), typically no strong criticism occurs (in fact, it’s sometimes viewed as “appropriate”, see Iyengar et al. 2019: 133). That is, the intergroup aversion, hierarchical thinking, and existence of social fault-lines at issue here seem generally more tolerated (Guimond et al. 2003). This doesn’t preclude the potential effectiveness of DDS-group specific social norms in constraining many of the intergroup biases highlighted above. But it does raise doubts about such norms’ efficacy.

Moreover, when the partial DD detection approach involves preventing the detection of invisible, individual-specific social identities, this might indeed reduce group members’ opportunities to develop tolerance towards these social identities. But notice that the approach is also explicitly aimed at *reducing* the harm (epistemic or otherwise) of social biases and stereotypes: just as the common procedure of blinding CVs so that applicant names can’t trigger unconscious gender and/or race biases skewing decision-making, ‘blinding’ DDS groups so that their members can’t detect certain social-identity cues of each other is meant to undercut interferences by unconscious biases and stereotypes and to facilitate egalitarian responding. That is, the partial DD detection approach in fact itself also helps directly counteract the pernicious influence of biases and stereotypes.

## Conclusion

I have argued that while DD can be epistemically beneficial even if it doesn’t involve CD, the epistemic benefits at issue depend on DD detection in different ways than assumed in recent work on diversity. Steel et al. (2019) suggest that DD detection might yield CD-independent epistemic benefits via triggering stereotype-based CD expectations. In response, I provided reasons to assume that DD detection implicating stereotyping is in fact likely to weaken epistemic group performance. Furthermore, while Carter and Phillips (2017) hold that DD detection can only result in CD-independent epistemic benefits if it involves social categorization specific enough for in-group vs. out-group distinctions and similarity-attraction, I argued against this. The kind of epistemic benefits at issue can result when group members only display partial DD detection, i.e., when they only have a general insight into the DD of their group such that they understand that the group is demographically diverse in many ways but can’t yet socially categorize each other with respect to their particular demographic features. Relating the distinction between specific and partial DD detection to scientific groups, I then proposed that in one common kind of demographically diverse groups in science, specific DD detection is likely to create epistemic costs. I suggested that, in contrast, partial DD detection helps avoid these costs and can also produce epistemic benefits. This point provides an epistemic reason for context-dependent limitations on scientists’ insight into the diversity of their group. While this reason is defeasible and may be outweighed by other considerations, it should be taken into account when analyzing the epistemic impact of diversity, especially when considering groups with invisible diversity in which members might remain largely hidden figures to each other.

## Footnotes

1 CD is also sometimes called ‘informational’, ‘functional’, or ‘task-related diversity’.

2 DD is also sometimes called ‘identity’ or ‘social category diversity’.

3 CD might be understood more broadly as simply capturing differences in perspectives, experiences, etc., i.e., without the additional point that these differences are also *task relevant*. We could then distinguish two kinds of CD: (i) CD involving task-relevant informational differences and (ii) CD involving task-irrelevant informational differences. However, to streamline the discussion, I shall here set (ii) aside and use the term ‘CD’ to specifically refer to CD (i) only. Having said that, it is worth noting (and will become clear in due course) that much of what I shall say below on the CD-independent epistemic benefits of DD carries over to CD (ii) too (i.e., there are CD (i)-independent epistemic benefits of CD (ii) too). The argument below can thus also be viewed as a challenge to much philosophical and social scientific research on CD which assumes what Page (2017) calls a “basic logic”: “For cognitive diversity to produce a bonus, it must be germane to the task” (169).

4 But *can* social categorization even occur without stereotyping? Carter and Phillips don’t support this assumption. It is, however, independently well motivated (Liberman et al. 2017). For instance, developmental psychologists found that “experimentally inducing essentialist beliefs about a novel social category […] did not lead” 4–6-year-olds to the “out-group dislike that defines social prejudice” (Rhodes et al. 2018: 1). Similarly, neuroscientific studies with adults measuring event-related brain potentials found a “dissociation between the perceptual encoding of race and subsequent activation of associated stereotypes” that indicated a “clear conceptual distinction between categorization and stereotyping” and suggests that the “encoding of racial category need not result in stereotype activation” (Ito and Tomelleri 2017: 758).

5 To argue that CD-independent epistemic benefits can be epistemically unjust, Steel et al. also consider other mechanisms that don’t involve stereotyping, for instance, that DD might counteract conformity in which people feel that members of their own identity group should be in agreement with one another. This mechanism doesn’t depend on stereotyping out-groups. My focus here is only on Steel et al.’s point concerning stereotyping, however.

6 According to Carter and Phillips, in a competitive context, social categorization should produce negative epistemic outcomes. Levine et al.’s experiment involved such a context; yet, it revealed positive outcomes. While the data challenge Carter and Phillips’ view, they are to be expected on the view proposed here: since subjects lacked specific insights into the distribution of DD in their groups, competition didn’t produce the negative effects tied to inter-group bias, out-group distrust, etc.

7 The assumption of informational differences of dissimilar others could also account for a potential diminished trust in dissimilar other. Crucially, such a reduced trust-attribution wouldn’t be epistemically unjust: it wouldn’t be unfair but simply overly cautious based on an exaggerated positive view of the interactants’ epistemic profile.

8 In some groups, specific DD detection is epistemically valuable by providing group members with cues about others’ expertise or reliability on certain issues (e.g., when in exploring AI-enhanced human cognition, one learns that a group member is both a psychologist and computer scientist). But the focus here is on DDS groups, and so the invisible aspects of DD at issue are (by assumption) task-irrelevant (see condition (d)). This means that specific DD detection of these aspects wouldn’t help group members to track relevant expertise among each other.
